# Vulnerable Plaque Is More Prevalent in Male Individuals at High Risk of Stroke: A Propensity Score-Matched Study

**DOI:** 10.3389/fphys.2021.642192

**Published:** 2021-04-09

**Authors:** Jie Li, Lijie Gao, Ping Zhang, Yingying Liu, Ju Zhou, Xingyang Yi, Chun Wang

**Affiliations:** ^1^Department of Neurology, People’s Hospital of Deyang City, Deyang, China; ^2^Department of Neurology, West China Hospital, Sichuan University, Chengdu, China

**Keywords:** gender, plaque vulnerability, risk factors, propensity score matching, atherosclerosis

## Abstract

**Objectives:**

To assess the gender differences in the prevalence of carotid vulnerable plaques in high-risk individuals for stroke in a multicenter, cross-sectional study.

**Methods:**

In the year 2015, 18595 residents who were at the age of 40 or older participated in a face-to-face study in eight communities in southwestern China. Totally 2,644 participants at high risk of stroke were enrolled. Before and after propensity score matching (PSM), the prevalence of carotid plaques and vulnerable plaques were compared between men and women. Multivariate analyses were applied to explore the association between the gender and carotid plaques. Stratified analyses and interaction tests were performed to identify factors that might modify the association between the gender and carotid plaques.

**Results:**

Among 2644 high-risk individuals enrolled, there were 1,202 (45.5%) men and 1442 (54.5%) women. Carotid plaques were detected in 904 (34.2%) participants, while vulnerable plaques were found in 425 (16.1%) participants. Before PSM, carotid plaques were more prevalent in male individuals than the female (36.7% vs. 32.1%, *p* = 0.01), as well as vulnerable plaque (20.0% vs. 12.8%, *p* < 0.01). Men tend to have a higher prevalence of vulnerable plaques in multivariate analyses (adjusted OR 1.70, 95% CI 1.10–2.62, *p* = 0.02). Stratified analyses and interaction tests demonstrated that the association between male sex and vulnerable carotid plaque did not change by age, family history of stroke, histories of chronic disease, smoking status, drinking status, physical activity, and BMI (all *p* for interaction > 0.05). After PSM, vulnerable plaques were still more prevalent in male individuals than the female (17.03% vs. 12.07%, *p* = 0.032).

**Conclusion:**

Male individuals had a higher risk of vulnerable carotid plaque independent of classical vascular risk factors. Whether there is a gender-specific association between variations in genes related to inflammation, lipid metabolis, and endothelial function and plaque vulnerability needs to be further studied.

## Introduction

Stroke is one of the leading causes of death and the major cause of adult disability worldwide, especially in China (GBD 2016 Causes of Death Collaborators, 2017; Wu et al., 2019). With the aging of the population, the onging high incidence of risk factors and inadequate management, the burden of stroke is increasing year by year (Wu et al., 2019). Approximately 80% of all strokes are ischemic and carotid artery atherosclerosis accounts for at least 20% of all ischemic strokes (Prasad, 2015; Puig et al., 2020).

Atherosclerosis is an chronic inflammatory disease of the arterial wall, with the characteristics of inflammation, endothelial injury, lipid accumulation, and extensive degradation of extracellular matrix components (Mangge and Almer, 2019; Wijeratne et al., 2020). Carotid atherosclerosis has been identified as a major risk factor of ischemic stroke, cardiovascular diseases, and other vascular events (Rundek et al., 2008; Sillesen et al., 2018; Parish et al., 2019). Ultrasound is a non-invasive and economical diagnostic technique that helps provide valuable information on carotid atherosclerosis such as carotid intima thickness (CIMT) and carotid plaque presence (Park, 2016). Several studies suggest that carotid plaque is more powerful in predicting vascular outcomes, compared with CIMT (Ho, 2016; Nezu and Hosomi, 2020).

Previous epidemiological researches have reported the associations between several classical vascular risk factors (such as age, hypertension, diabetes, dyslipidemia, and current smoking) and carotid plaques (Sturlaugsdottir et al., 2016; Bian et al., 2018; Noflatscher et al., 2019; Santos-Neto et al., 2021). It is noted that the incidence of stroke is higher in male individuals compared with the female age < 75 years (Lloyd-Jones et al., 2010), and gender differences in plaque characteristics might help explain this phenomenon. However, there is scarce information available about the gender differences in the prevalence of carotid plaque in high-risk individuals for stroke. Previous studies which investigated the association between sex and intra-plaque hemorrhage (IPH) mainly focused on patients with moderate or severe carotid stenosis (Ota et al., 2010). Meanwhile, the judgment of IPH in carotid plaque was mainly based on histopathological examination after carotid endarterectomy (CEA) (Hellings et al., 2007). Therefore, we conducted the present study using the data of a multicenter, cross-sectional survey in China to explore the gender differences in the prevalence of carotid plaques among individuals at high risk of stroke.

## Materials and Methods

### Study Design and Participants

The present study was a branch of the China National Stroke Screening Survey (CNSSS) program of the National Health and Family Planning Commission of China (grant No. 2011BAI08B01) (Li et al., 2015; Yi et al., 2020a,b). The CNSSS which aimed to provide stroke prevention policies for the Chinese, is a population-based cross-sectional study with a 2-stage stratified sampling framework (Li et al., 2015; Yi et al., 2020a,b). More details of the CNSSS could be followed at the official website (Stroke Prevention Project Committee, 2018). From May 1, 2015 to Sep 31, 2015, the present study was conducted in eight randomly selected communities of Sichuan province in southwestern China, using a cluster survey method (Yi et al., 2020a,b). This survey was performed among residents aged ≥ 40 and who lived more than 6-month in each community. Ethics Committee of the three participating institutions (People’s Hospital of Deyang City, Affiliated Hospital of Southwest Medical University, the Suining Central Hospital) approved our study protocol and written informed consent was obtained from all participants enrolled in this study (Yi et al., 2020a,b).

### Data Collection and the Definition of High-Risk Individuals for Stroke

Data were collected via using a standardized structured face-to-face questionnaire by experienced surveyors, including demographic information (age, sex, education level), family history of stroke, behavior factors (smoking, drinking, exercise habits), history of stroke [ischemic stroke or transient ischemic attack (TIA), hemorrhagic stroke], history of chronic diseases (hypertension, dyslipidemia, diabetes mellitus, and atrial fibrillation) (Yi et al., 2020a,b). Body measurements of height, weight, waist circumference, and hip circumference were also measured and recorded in the questionnaire. The eight stroke-related risk factors were assessed, including hypertension, dyslipidemia, diabetes mellitus, atrial fibrillation, current smoking (≥1 cigarette per day), physical inactivity (physical exercise < 3 times per week for < 30 min each time), overweight/obesity [defined as body mass index (BMI) ≥ 26 kg/m^2^], and a family history of stroke, which has been elaborated upon in our previous study (Yi et al., 2020a,b). Participants who had at least three of the above eight risk factors or had a history of stroke were identified as the high-risk participants for stroke (Wang et al., 2017).

Laboratory examinations such as fasting blood glucose (FBG), hemoglobin A1c, triglycerides, total cholesterol (TC), high-density lipoprotein cholesterol (HDL-C), low-density lipoprotein cholesterol (LDL-C), and homocysteine, electrocardiogram (ECG), and carotid ultrasonography were also obtained from the high-risk participants for stroke (Yi et al., 2020a,b). Detailed methods for data collection have been elaborated upon in our previous studies (Yi et al., 2020a,b).

### Carotid Ultrasound Examination

Diagnostic ultrasound (type 512, ACUSON Sequoia Apparatus, 7.5 MHz probe, Berlin, Germany) was performed in participants at high risk of stroke to assessed bilateral common and internal carotid arteries, as well as bifurcations according to standard scanning reading protocols (Rundek et al., 2008; Yi et al., 2020b). Detailed procedures for evaluating the characteristics of carotid plaque have been described in our previous study (Yi et al., 2016, 2017, 2020b). An atherosclerotic plaque was defined as the presence of an endoluminal protrusion > 1.5 mm or a focal thickening at least 50% greater of the CIMT than adjacent arterial wall (Rundek et al., 2008; Yi et al., 2020b). Based on the plaque echogenicity and surface appearance, carotid plaques were further classified from class I to class IV as uniformly echolucent, predominantly echolucent, predominantly echogenic, and echogenic, respectively (Mathiesen et al., 2001; Yi et al., 2020b). Plaques of class I or II were identified as vulnerable plaques, while plaques of class III or IV were identified as stable plaques (Yi et al., 2016, 2017, 2020b). Carotid plaques were independently classified by ultrasound practitioners who were blinded to baseline information.

### Statistical Analyses

Clinical characteristics are presented as means with standard deviations (SDs) for continuous variables and as frequencies with percentages for categorical variables according to different genders. Intergroup differences in categorical variables were calculated for significance using the χ^2^-tests or Fisher’s exact tests, while intergroup differences in continuous variables were calculated using the Student’s *t*-tests or Mann-Whitney *U*-test (Li et al., 2020a).

Univariate analysis comparing factors associated with carotid plaque and vulnerable plaque in high-risk individuals for stroke was performed. Multivariate logistic regression was performed to identify the association between gender and carotid plaque in high-risk individuals in 4 different models. Model 1 was adjusted for age and family history of stroke. Model 2 was adjusted for variables in model 1 + BMI. Model 3 was adjusted for variables in model 1 + BMI + vascular risk factors (history of ischemic stroke or TIA, hypertension, dyslipidemia, diabetes mellitus, smoking status). Model 4 was adjusted for variables in model 1 + BMI + vascular risk factors + laboratory test (Hemoglobin A1c, FBG, Triglycerides, TC, HDL-C, LDL-C). Stratified analyses and interaction tests were conducted according to age, family history of stroke, histories of chronic disease, smoking status, drinking status, physical activity, and BMI, to identify factors that might modify the association between the gender and carotid plaques. The significance of interaction was tested by the log-likelihood ratio test.

We also performed a propensity score matching (PSM) algorithm including baseline characteristics that are assumed to be related to the gender by using a multivariate logistic regression analysis, to calculate the propensity score for each patient. Then participants between different gender groups were matched via using the nearest neighbor approach (caliper 0.2, ratio 1:1) to minimize potential imbalances between the two groups as previously described in detail (Li et al., 2020a). Gender differences in the prevalence of carotid plaques and vulnerable plaques were compared before and after PSM.

The 95% confidence intervals (CI) were calculated to describe the precision of the estimates. Two-sided *P* < 0.05 was considered statistically significant for all results. All statistical analyses were performed using SPSS 21.0 software (IBM, Chicago, IL, United States), statistical software packages R (The R Foundation, version 3.4.3)^1^ and EmpowerStats (X&Y Solutions, Inc., Boston, MA, United States)^2^, which have been described in our previous studies (Li et al., 2020a,b).

## Results

**FIGURE 1:**
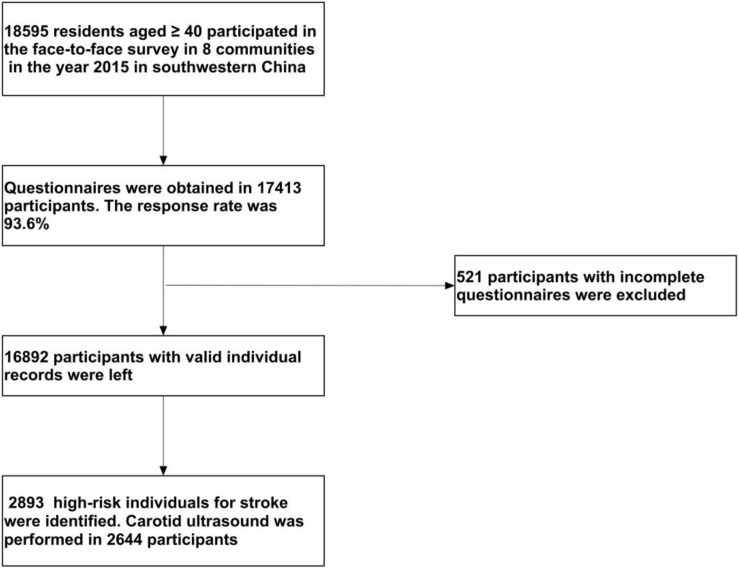
Flow diagram of the data preparing and cleaning process in this survey.

In the year 2015, 18595 residents aged ≥ 40 participated in the face-to-face survey in eight communities in Sichuan province in southwestern China. Finally, a total of 2644 subjects at high risk of stroke were enrolled, comprising 1,202 men and 1,442 women aged 63.3 ± 9.8 years. A flow diagram of the data preparing and cleaning process in this survey is provided in Figure 1.

### Gender Differences in the Characteristics of High-Risk Individuals and the Prevalence of Carotid Plaque

Gender differences in the characteristics of high-risk individuals and the prevalence of carotid plaque were exhibited in Table 1. Compared with women, men were younger (62.7 ± 10.3 vs. 63.7 ± 9.4 years, *p* < 0.01), had higher educational level (*p* < 0.01), more history of former and current smoking (13.9%, 54.2% vs. 1.7%, 4.4%, respectively, *p* < 0.01) and regular alcohol consumption (18.7% vs. 1.6%, *p* < 0.01), less history of ischemic stroke or transient ischemic stroke (TIA) (14.4% vs. 20.5%, *p* < 0.01), diabetes (28.4% vs. 39.3%, *p* < 0.01), dyslipidemia (67.8% vs. 76.5%, *p* < 0.01) and atrial fibrillation (7.8% vs. 11.0%, *p* < 0.01). Meanwhile, the level of FBG (6.3 ± 2.2 vs. 6.5 ± 2.7 mmol/L, *p* = 0.02), total cholesterol (5.1 ± 1.0 vs. 5.3 ± 1.0 mmol/L, *p* < 0.01), HDL-C (1.5 ± 0.5 vs. 1.7 ± 0.6 mmol/L, *p* < 0.01) and LDL-C (2.9 ± 0.9 vs. 3.1 ± 0.9 mmol/L, *p* < 0.01) were lower in men than in women in the current survey. However, men had a higher level of homocysteine than women (15.5 ± 11.1 vs. 11.9 ± 7.2 mmol/L, *p* < 0.01). Carotid plaques were found in 904 (34.2%) subjects, and 425 (16.1%) had vulnerable plaques. The total prevalence of carotid plaque was higher in men than in women (36.7% vs. 32.1%, *p* = 0.01), as well as vulnerable plaque (20.0% vs. 12.8%, *p* < 0.01).

**TABLE 1:** Gender differences in the characteristics of individuals at high risk of stroke and the prevalence of carotid plaque.

Variables	Total (*n* = 2,644)	Male (*n* = 1,202)	Female (*n* = 1,442)	*P-*value
Age, year, mean ± SD	63.3 ± 9.8	62.7 ± 10.3	63.7 ± 9.4	<0.01*
Education, n (%)				<0.01^‡^
Primary school or below	1833 (69.3)	762 (63.4)	1071 (74.3)	–
Junior middle school	658 (24.9)	343 (28.5)	315 (21.8)	–
Senior middle school	120 (4.5)	73 (6.1)	47 (3.3)	–
College or above	33 (1.3)	24 (2.0)	9 (0.6)	–
Family history of stroke, n (%)	474 (17.9)	206 (17.1)	268 (18.6)	0.33^‡^
Vascular risk factors, n (%)				
Ischemic stroke or TIA	468 (17.7)	173 (14.4)	295 (20.5)	<0.01^‡^
Hemorrhagic stroke	93 (3.5)	36 (3.0)	57 (4.0)	0.18^‡^
Hypertension	2122 (80.3)	945 (78.6)	1177 (81.6)	0.05^‡^
Diabetes mellitus	907 (34.3)	341 (28.4)	566 (39.3)	<0.01^‡^
Dyslipidemia	1918 (72.5)	815 (67.8)	1103 (76.5)	<0.01^‡^
Atrial fibrillation	252 (9.5)	94 (7.8)	158 (11.0)	<0.01^‡^
Smoking status, n (%)				<0.01^‡^
Never	1736 (65.7)	383 (31.9)	1353 (93.8)	–
Former	192 (7.3)	167 (13.9)	25 (1.7)	–
Current	716 (27.1)	652 (54.2)	64 (4.4)	–
Alcohol consumption, n (%)	248 (9.4)	225 (18.7)	23 (1.6)	<0.01^‡^
Physical inactivity, n (%)	1698 (64.2)	757 (63.0)	941 (65.3)	0.22^‡^
Overweight or obesity, n (%)	1647 (62.3)	744 (61.9)	903 (62.6)	0.69^‡^
BMI, kg/m	26.0 ± 3.6	25.9 ± 3.3	26.1 ± 3.7	0.16*
Waist circumference, cm	88.9 ± 12.0	88.9 ± 10.0	86.4 ± 11.6	<0.01*
Hip circumference, cm	95.2 ± 11.9	95.9 ± 10.5	94.6 ± 13.0	0.16*
Hemoglobin A1c, mmol/L	6.8 ± 1.8	6.9 ± 2.0	6.6 ± 1.6	<0.01*
FBG, mmol/L	6.4 ± 2.5	6.3 ± 2.2	6.5 ± 2.7	0.02*
Total cholesterol, mmol/L	5.2 ± 1.0	5.1 ± 1.0	5.3 ± 1.0	<0.01*
HDL-C, mmol/L	1.6 ± 0.6	1.5 ± 0.5	1.7 ± 0.6	<0.01*
LDL-C, mmol/L	3.0 ± 0.9	2.9 ± 0.9	3.1 ± 0.9	<0.01*
Triglycerides, mmol/L	1.8 ± 1.9	1.8 ± 2.2	1.8 ± 1.6	0.36*
Homocysteine, mmol/L	13.6 ± 9.4	15.5 ± 11.1	11.9 ± 7.2	<0.01*
Total carotid plaque, n (%)	904 (34.2)	441 (36.7)	463 (32.1)	0.01^‡^
Vulnerable carotid plaque, n (%)	425 (16.1)	240 (20.0)	185 (12.8)	<0.01^‡^

Data are presented as mean ± SD, median (range), or number (%).

*Student t-test. ^‡^χ2 test.

*BMI, Body mass index; FBG, fasting blood glucose; HDL-C, high density lipoprotein cholesterol; LDL-C, low density lipoprotein cholesterol*.

### Risk Factors Associated With Carotid Plaques in High-Risk Individuals for Stroke

Univariable analysis of risk factors associated with total carotid plaques and vulnerable plaques was presented in Table 2. Male sex was associated with both carotid plaque (OR 1.23, 95%CI 1.04–1.44, *p* = 0.01) and vulnerable plaque (OR 1.70, 95%CI 1.37–2.09, *p* < 0.01). Besides, age, history of ischemic stroke/TIA, hypertension, dyslipidemia, former or current smoking, baseline hemoglobin A1c, FBG, TC, and LDL-C were associated with total carotid plaque (all *p* < 0.05). Meanwhile, age, hypertension, former or current smoking, baseline hemoglobin A1c, FBG, triglycerides, TC, and LDL-C were associated with vulnerable plaque (all *p* < 0.05).

**TABLE 2:** Univariable analysis for the factors associated with carotid plaques in a population at high risk of stroke.

Variables*	Total carotid plaque		Vulnerable carotid plaque	
		
	OR (95%CI)	*P-*value	OR (95%CI)	*P-*value
Age, year	1.07 (1.06–1.08)	<0.01	1.05 (1.04–1.06)	<0.01
Male	1.23 (1.04–1.44)	0.01	1.70 (1.37–2.09)	<0.01
Education level				
College or above	Reference	–	Reference	–
Primary school or below	1.22 (0.59–2.52)	0.60	1.56 (0.55–4.48)	0.41
Junior middle school	0.65 (0.31–1.38)	0.26	0.98 (0.33–2.85)	0.96
Senior middle school	0.93 (0.41–1.10)	0.86	1.28 (0.40–4.08)	0.68
Family history of stroke	0.82 (0.66–1.02)	0.07	0.77 (0.57–1.02)	0.07
Vascular risk factors				
Ischemic stroke or TIA	0.71 (0.57–0.88)	<0.01	0.88 (0.67–1.17)	0.39
Hemorrhagic stroke	1.41 (0.93–2.14)	0.11	1.09 (0.63–1.88)	0.76
Hypertension	1.67 (1.35–2.07)	<0.01	1.64 (1.22–2.19)	<0.01
Dyslipidemia	1.22 (1.02–1.47)	0.03	1.20 (0.95–1.53)	0.13
Diabetes mellitus	1.06 (0.90–1.26)	0.50	1.09 (0.88–1.36)	0.42
Atrial fibrillation	1.12 (0.85–1.47)	0.42	1.02 (0.71–1.44)	0.93
Smoking status				
Never	Reference	–	Reference	–
Former	2.04 (1.51–2.75)	<0.01	1.93 (1.34–2.77)	<0.01
Current	1.36 (1.13–1.63)	<0.01	1.70 (1.36–2.14)	<0.01
Alcohol consumption	1.13 (0.86–1.48)	0.38	1.11 (0.78–1.56)	0.57
Physical inactivity	1.00 (0.84–1.18)	0.96	0.92 (0.74–1.14)	0.44
BMI	1.00 (0.98–1.02)	0.99	0.97 (0.95–1.00)	0.09
Waist circumference	1.00 (0.99–1.01)	0.38	1.00 (0.99–1.01)	0.85
Hip circumference	1.00 (0.98–1.01)	0.78	0.99 (0.97–1.01)	0.38
laboratory test				
Hemoglobin A1c	1.08 (1.01–1.16)	0.03	1.09 (1.01–1.18)	0.03
FBG	1.04 (1.01–1.08)	0.01	1.05 (1.01–1.09)	0.01
Triglycerides	1.01 (0.97–1.05)	0.71	1.05 (1.01–1.11)	0.03
TC	1.19 (1.10–1.29)	<0.01	1.21 (1.10–1.33)	<0.01
HDL-C	1.20 (1.04–1.37)	0.01	1.05 (0.88–1.24)	0.59
LDL-C	1.22 (1.11–1.34)	<0.01	1.26 (1.12–1.42)	<0.01
Homocysteine	1.01 (1.00–1.02)	0.06	1.00 (0.99–1.01)	0.72

*OR, odds ratio; CI, confidence intervals; BMI, Body mass index; FBG, fasting blood glucose; TC, total cholesterol; HDL-C, high density lipoprotein cholesterol; LDL-C, low density lipoprotein cholesterol*.

### The Association Between Male Sex and Carotid Plaque in High-Risk Individuals for Stroke

As presented in Table 3, multivariate logistic regression was conducted to explore the association between male sex and total carotid plaque or vulnerable plaque. After adjusting for age, family history of stroke, and BMI (model 1 or 2), male sex was significantly associated with total carotid plaque (*p* < 0.01) and vulnerable plaque (*p* < 0.01). When vascular risk factors (including a history of ischemic stroke or TIA, hypertension, dyslipidemia, diabetes mellitus, smoking status) and lab tests (including hemoglobin A1c, FBG, triglycerides, TC, HDL-C, LDL-C) were included in the multivariate logistic regression (model 3 or 4), male sex was no longer an independent risk factor for carotid plaque, however, the male was still an independent risk factor for vulnerable plaque (adjusted OR 1.70, 95%CI 1.10–2.62, *p* = 0.02, in model 4) than female.

**TABLE 3:** Multiple logistic regression analysis for the association between male sex and carotid plaque in a population at high risk of stroke.

	Total carotid plaque		Vulnerable carotid plaque	
		
	OR (95%CI)	*P-*value	OR (95%CI)	*P-*value
Unadjusted	1.23 (1.04–1.44)	0.01	1.70 (1.37–2.09)	<0.01
Model 1	1.33 (1.12–1.58)	<0.01	1.79 (1.45–2.22)	<0.01
Model 2	1.35 (1.14–1.60)	<0.01	1.79 (1.45–2.22)	<0.01
Model 3	1.03 (0.82–1.29)	0.82	1.49 (1.13–1.97)	<0.01
Model 4	1.30 (0.89–1.89)	0.17	1.70 (1.10–2.62)	0.02

*Model 1 was adjusted for age and family history of stroke.*

*Model 2 was adjusted for variables in model 1 + BMI.*

*Model 3 was adjusted for variables in model 1 + BMI + vascular risk factors (history of ischemic stroke or TIA, hypertension, dyslipidemia, diabetes mellitus, smoking status).*

*Model 4 was adjusted for variables in model 1 + BMI + vascular risk factors + laboratory test (Hemoglobin A1c, FBG, Triglycerides, TC, HDL-C, LDL-C).*

*OR, odds ratio; CI, confidence intervals; BMI, Body mass index; FBG, fasting blood glucose; TC, total cholesterol; HDL-C, high density lipoprotein cholesterol; LDL-C, low density lipoprotein cholesterol.*

### Stratified Analyses and Interaction Test of the Association Between Male Sex and Vulnerable Plaque

**FIGURE 2:**
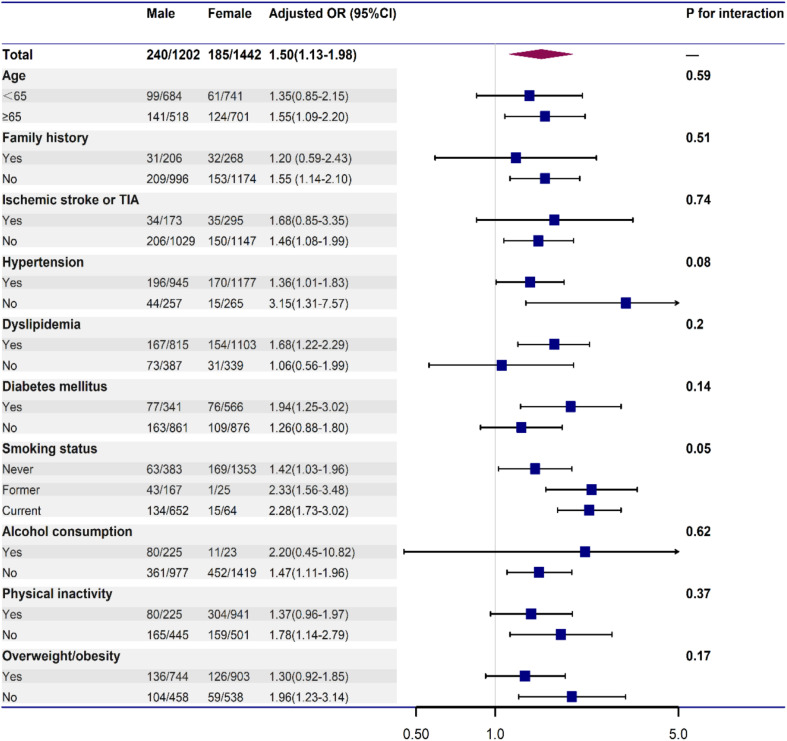
Stratified logistic regression analysis to identify variables that modify the association between male and vulnerable carotid plaque. Each stratification was adjusted for age, family history of stroke, histories of chronic disease (ischemic stroke or TIA, hypertension, dyslipidemia, diabetes mellitus), smoking status, regular alcohol consumption, physical inactivity, and BMI, except for the stratification factor itself.

To further explore the association between male sex and vulnerable carotid plaques, stratified analyses and interaction tests were employed. In Figure 2, we found that the association between male sex and vulnerable carotid plaque did not change by age, family history of stroke, histories of chronic disease (ischemic stroke or TIA, hypertension, dyslipidemia, diabetes mellitus), smoking status, drinking status, physical activity and BMI (all *p* for interaction > 0.05). Male individuals tended to have a stronger association with vulnerable carotid plaque compared with the female, as shown in Table 3.

### Gender Differences in the Prevalence of Carotid Plaque and Vulnerable Plaque After PSM

After PSM, we identified two subgroups of 928 participants (including 464 men and 464 women) at high risk of stroke that were balanced for all characteristics. Relative multivariate imbalance in terms of the L1 measure was smaller (0.468 vs. 0.733) and no covariate had standardized mean differences > 0.1 after PSM. As shown in Table 4, there was no significant difference in the characteristics between different gender groups in the matched dataset (all *p* > 0.05). After PSM, men no longer had more carotid plaque (33.2% vs. 31.9%, *p* = 0.67), however, vulnerable plaques were still more prevalent in male individuals (17.0% vs. 12.1%, *p* = 0.03).

**TABLE 4:** Gender differences in the characteristics of individuals at high risk of stroke and the prevalence of carotid plaque after PSM.

Variables	Total (*n* = 928)	Male (*n* = 464)	Female (*n* = 464)	*P*-value
Age, year, mean ± SD	63.1 ± 10.0	63.2 ± 10.4	63.0 ± 9.7	0.77*
Education, n (%)				0.215^‡^
Primary school or below	620 (66.8)	297 (64.0)	323 (69.6)	–
Junior middle school	256 (27.6)	136 (29.3)	120 (25.9)	–
Senior middle school	42 (4.5)	26 (5.6)	16 (3.5)	–
College or above	10 (1.1)	5 (1.1)	5 (1.1)	–
Family history of stroke, n (%)	179 (19.3)	85 (18.3)	94 (20.3)	0.45^‡^
Vascular risk factors, n (%)				
Ischemic stroke	151 (16.3)	72 (15.5)	79 (17.0)	0.53^‡^
Hemorrhagic stroke	33 (3.6)	19 (4.1)	14 (3.0)	0.38^‡^
Hypertension	763 (82.2)	383 (82.5)	380 (81.9)	0.80^‡^
Diabetes mellitus	328 (35.3)	158 (34.1)	170 (36.6)	0.41^‡^
Dyslipidemia	651 (70.2)	332 (71.6)	319 (68.8)	0.35^‡^
Atrial fibrillation	89 (9.6)	47 (10.1)	42 (9.1)	0.58^‡^
Smoking status, n (%)				0.28^‡^
Never	750 (80.8)	375 (80.8)	375 (80.8)	
Former	41 (4.4)	16 (3.5)	25 (5.4)	
Current	137 (14.8)	73 (15.7)	64 (13.8)	
Alcohol consumption, n (%)	51 (5.5)	29 (6.3)	22 (4.7)	0.31^‡^
Physical inactivity, n (%)	587 (63.3)	285 (61.4)	302 (65.1)	0.25^‡^
Overweight or obesity, n (%)	595 (64.1)	301 (64.9)	294 (63.4)	0.63^‡^
BMI, kg/m2	26.5 ± 3.5	26.2 ± 3.3	26.3 ± 3.8	0.53*
Waist circumference, cm	89.2 ± 8.9	87.0 ± 11.2	86.6 ± 10.6	0.61*
Hip circumference, cm	95.2 ± 11.0	95.9 ± 9.8	94.5 ± 12.2	0.34*
FBG, mmol/L	6.6 ± 2.8	6.5 ± 2.7	6.7 ± 2.9	0.28*
Total cholesterol, mmol/L	5.2 ± 1.1	5.2 ± 1.1	5.3 ± 1.1	0.30*
HDL-C, mmol/L	1.7 ± 0.7	1.7 ± 0.7	1.7 ± 0.7	0.63*
LDL-C, mmol/L	3.0 ± 0.8	2.9 ± 0.9	3.0 ± 0.8	0.80*
Triglycerides, mmol/L	1.7 ± 1.6	1.7 ± 1.6	1.7 ± 1.8	0.90*
Homocysteine, mmol/L	7.8 ± 1.3	7.8 ± 1.2	7.8 ± 1.3	0.93*
Total carotid plaque, n (%)	302 (32.5)	154 (33.2)	148 (31.9)	0.67^‡^
Carotid vulnerable plaque, n (%)	135 (14.6)	79 (17.0)	56 (12.1)	0.03^‡^

Data are presented as mean ± SD, median (range), or number (%).

*Student t-test. ^‡^χ^2^-test.

*BMI, Body mass index; FBG, fasting blood glucose; HDL-C, high density lipoprotein cholesterol; LDL-C, low density lipoprotein cholesterol*.

## Discussion

Atherosclerosis in the carotid artery can lead to plaque vulnerability, which is one of the main causes of ischemic stroke (Howard et al., 2015; Pelisek et al., 2012). Our present study have identified a high prevalence of total carotid plaque (34.2%) and vulnerable carotid plaque (16.1%) among high-risk participants for stroke in southwestern China and demonstrated that male individuals have a higher risk of vulnerable plaques than the female (adjusted OR 1.70, 95% CI 1.10–2.62), even after propensity score -matched. Moreover, stratified analyses and interaction tests showed that the stronger association between male sex and vulnerable plaque did not change by age, family history of stroke, histories of chronic disease (ischemic stroke or TIA, hypertension, dyslipidemia, diabetes mellitus), smoking status, drinking, physical activity, and BMI, suggesting that male is associated with a higher risk of vulnerable plaque independent of classical vascular risk factors.

It has been demonstrated that age, hypertension, diabetes, high low-density lipoprotein cholesterol levels, and current smoking are traditional cardiovascular risk factors related to the prevalence of carotid plaques (Sturlaugsdottir et al., 2016; Bian et al., 2018; Noflatscher et al., 2019; Santos-Neto et al., 2021). However, there is scarce information regarding the gender differences in the prevalence of carotid plaques in participants at high risk of stroke, especially vulnerable plaque. It is known that IPH is one of the major characteristics of vulnerable plaque, several researchers have investigated the association between sex and IPH in the carotid artery (Hellings et al., 2007; Ota et al., 2010; Vrijenhoek et al., 2013). Observational research based on histological analysis of CEA specimens found that female individuals tend to have a more stable, less inflammatory carotid plaques compared with the male, independent of clinical manifestation and cardiovascular risk factors (Hellings et al., 2007). Similarly, a cohort study conducted in patients who had undergone CEA suggested that carotid plaques obtained from male individuals had a higher prevalence of IPH compared with the female (Vrijenhoek et al., 2013). Another study enrolled patients with asymptomatic moderate or severe carotid stenosis, and suggested that men had more high-risk plaques compared with women after justing for potential confounders (Ota et al., 2010). Our study demonstrated that male individuals had a higher risk of vulnerable carotid plaques than the females, which is similar to the results of previous studies (Hellings et al., 2007; Ota et al., 2010; Vrijenhoek et al., 2013). The difference between our study and previous studies is that the current study was a population-based study conducted in high-risk individuals for stroke, which is different from previous studies that mainly focused on patients with moderate or severe carotid stenosis, even posttreatment of CEA.

The underlying pathophysiologic mechanisms that explain these gender differences of the prevalence of vulnerable carotid plaque are poorly understood. There are several possible reasons for this. First, although men do not experience a rapid decline in endogenous sex hormone production, an age-related decrease in the levels of endogenous sex hormone especially testosterone might have an important effect on the progression of atherosclerosis. It has been demonstrated that low levels of free testosterone are associated with the progression of carotid atherosclerosis in elderly men independently of classical vascular risk factors (Muller et al., 2004; Svartberg et al., 2006; Soisson et al., 2012). Second, there are differences in the protective effect of estrogen on atherosclerosis between the two genders (Yahagi et al., 2015). Estrogen might play a direct effect on matrix metalloproteinase production contributing to the attenuation of atherosclerotic disease in females (Yahagi et al., 2015). A recently published observational study found that men have more age-specific carotid IPH in magnetic resonance imaging compared with women. However, among post-menopausal women, the risk of carotid IPH becomes closer to that of men with increasing age (Singh et al., 2017). It has been found that men with the common genetic variation in estrogen receptor alpha have three times higher risk of myocardial infarction as compared to those without variant (Shearman et al., 2003), which indicates that genetic factors might play an essential role in the gender differences of atherosclerosis. A previous study found that only 19.5% of the carotid plaque burden could be explained by traditional and less traditional vascular risk factors, also suggesting that genetic and environmental factors might play a major role in the determination of atherosclerosis (Kuo et al., 2012). Until recently, variation in genes related to inflammation, endothelial function, and lipid metabolism are thought to be linked to carotid plaque burden (Gardener et al., 2011; Wang et al., 2011; Stroke Prevention Project Committee, 2018). Whether there is a gender-specific association between variations in genes related to inflammation, endothelial function, and lipid metabolism and plaque vulnerability has not been adequately studied.

### Limitations

The results of the present study should be interpreted with caution given its limitations. First, although a standardized structured face-to-face questionnaire was used by experienced surveyors to collect data including demographic characteristics, behavior factors, family history of stroke, history of stroke and chronic disease, and physical examination, the application of the self-reported questionnaire might also be associated with recall bias and make the answers unreliable. Second, even though we conduct a multicenter population-based study with a large number of subjects recruited and the large number of variables collected, we only screened residents ages ≥ 40 years and we did not compare the gender differences of carotid plaque in residents who were not identified as the high-risk individuals for stroke, therefore, our results might not represent the whole population. Third, carotid plaque and plaque vulnerability were evaluated by carotid ultrasound but not high-resolution magnetic resonance imaging, which could provide more information including plaque composition and morphology. Besides, the data collection was done many years ago and this is unlikely to provide an updated picture of the situation. Furthermore, we did not explore the effect of antiplatelet drugs or statins on plaque vulnerability in our study due to a lack of data. Finally, limited to the study protocol of the CNSSS program, we could not provide information related to inflammatory markers such as the level of C-reactive protein or other acute-phase protein, and further studies are needed to explore this issue.

## Conclusion

Despite the above limitations, this multicenter, cross-sectional study provides clear evidence that male individuals had a higher risk of vulnerable carotid plaque independent of classical vascular risk factors, genetic factors might play a major role in the gender differences in the progression of atherosclerosis. Whether there is a gender-specific association between variations in genes involved in inflammation, endothelial function, and lipid metabolism and plaque vulnerability needs to be further studied.

## Data Availability Statement

The raw data supporting the conclusions of this article will be made available by the corresponding author on reasonable request.

## Ethics Statement

Our study protocol was approved by the ethics committee of three participating hospitals (the People’s Hospital of Deyang City, the Affiliated Hospital of Southwest Medical University, and the Suining Central Hospital). Informed consents were obtained from all participants during recruitment. The patients/participants provided their written informed consent to participate in this study.

## Author Contributions

JL and LG collected, analyzed, and interpreted the data, as well as drafted the manuscript. PZ, YL, and JZ participated in study conception and design, data interpretation, and revised the manuscript. XY and CW contributed substantially to study design and supervision, data interpretation, and manuscript writing. All authors critically revised the manuscript for important intellectual content and approved the final manuscript.

## Conflict of Interest

The authors declare that the research was conducted in the absence of any commercial or financial relationships that could be construed as a potential conflict of interest.

## Footnotes

1 http://www.R-project.org

2 http://www.empowerstats.com
